# Effects of Individual Dietary Intervention on Nutrient Intake in Postpartum Japanese Women: A Randomized Controlled Trial

**DOI:** 10.3390/nu13093272

**Published:** 2021-09-19

**Authors:** Mie Shiraishi, Masayo Matsuzaki, Rina Tsunematsu, Sachi Watanabe, Risa Kobayashi, Megumi Haruna

**Affiliations:** 1 Department of Midwifery and Women’s Health, Division of Health Sciences and Nursing, Graduate School of Medicine, The University of Tokyo, Tokyo 113-0033, Japan; matsu3@sahs.med.osaka-u.ac.jp (M.M.); nigiir79@gmail.com (R.T.); sachi5683@tempo.ocn.ne.jp (S.W.); risaaaaan115@gmail.com (R.K.); mharuna@m.u-tokyo.ac.jp (M.H.); 2 Department of Children and Women’s Health, Division of Health Sciences, Graduate School of Medicine, Osaka University, Osaka 565-0871, Japan

**Keywords:** breastfeeding, food, nutrition assessment, postpartum period, randomized controlled trial

## Abstract

No dietary intervention that focuses on the diet quality of postpartum women has been developed in Japan, although most postpartum women experience an insufficient intake of vitamins and minerals. We aimed to examine whether dietary intervention, based on the health belief model, at both 1 and 3 months postpartum affects nutrient intake and food group consumption at 6 months postpartum. A randomized controlled trial was conducted at a university hospital in Tokyo between 2015 and 2016. Healthy women at 1 month postpartum were randomly allocated to either an intervention group (*n* = 100) or a control group (*n* = 94). Dietary intervention included dietary assessment, individual feedback, and dietary guidance. The dietary intakes between the two groups were compared using the Mann-Whitney *U* test. At 6 months postpartum, the energy-adjusted intakes of protein, total dietary fiber, potassium, magnesium, phosphorus, zinc, vitamin B_6_, and β-carotene were significantly higher in the intervention group than in the control group. The changes in energy-adjusted intakes of total dietary fiber and iron from 1 month postpartum to 6 months postpartum were significantly different between the two groups. Dietary intervention based on the health belief model improved nutrition at 6 months postpartum, although the impact was limited.

## 1. Introduction

Adequate nutrition is essential during the months following childbirth because the mother is recovering from childbirth and any nutrients consumed by the mother pass to the baby through breast milk [1]. Nevertheless, 50–90% of women in the year following childbirth have insufficient intake of important nutrients, such as protein, calcium, iron, vitamin D, and folate in Japan, although more than 70% reported that they paid necessary attention to the need for adequate nutrition [2,3]. Inadequate nutritional status during the postpartum period is associated with certain diseases; for instance, iron deficiency commonly causes persistent anemia. Indeed, iron deficiency anemia diagnosed in the early postpartum period was found to be sustained in 21% of women at 1 month postpartum [4]. Furthermore, in lactating women, a certain amount of iron is transferred to babies through breast milk, irrespective of the mother’s iron intake [5]; thus, lactating women with low iron intake are more likely to have iron deficiency.

The majority of postpartum women in Japan do not receive any dietary guidance since individual dietary assessment is not a component of the hospital stay after childbirth and standard medical checkups during the postpartum period [2]. Nevertheless, postpartum women, particularly those who are breastfeeding, often feel anxious that their nutrient intake might be excessive or deficient with regard to their breast milk and weight retention and need proper information on nutrition during this period [2,6]. Thus, greater understanding of dietary intake may lead to dietary behavioral changes.

In Western countries, several dietary education interventions for weight loss and diabetes mellitus in the postpartum period have been developed [7,8,9,10]. Among the studies that focused on dietary habits and quality, only one study reported significantly improved diet quality, such as reduction in confectionery and increase in vegetable intake [9]. In Japan, although no intervention for postpartum women has yet been developed, a prenatal intervention based on the health belief model (HBM) was recently developed to improve diet quality and food choices [11]. The prenatal intervention was successful in increasing circulating levels of eicosapentaenoic acids, docosahexaenoic acids, and vitamin D [11]. The HBM is an interpersonal model that explains and predicts health-related behaviors by incorporating perceived susceptibility and severity of diseases, perceived benefits and barriers to healthier behaviors, cues to action, and self-efficacy [12,13,14]. Based on these components, the prenatal dietary intervention included dietary assessment, individual feedback, and information on the risk of nutrient deficiencies [11]. Such an intervention might have helped pregnant women understand the risk of nutrition-related diseases and subsequently improve their prenatal dietary habits. Likewise, individual dietary intervention based on the HBM in the postpartum period may also be effective in improving diet quality.

The present study aimed to examine whether dietary intervention, including individual dietary assessment and feedback and providing dietary information for women at 1 and 3 months postpartum, affects the intake of nutrients and food groups at 6 months postpartum.

## 2. Materials and Methods

### 2.1. Study Design

A randomized control trial was conducted in a university hospital in Tokyo, Japan, between June 2015 and July 2016. Participants were allocated 1:1 to either an intervention group (IG) or a control group (CG) using concealed random allocation from a computer-generated random numbers table. The research secretary managing the table followed a simple up-to-down method for participant allocation and provided the researchers with the respective intervention allocation and serial ID number at the time of recruitment. This study did not blind the participants due to the fact that it was a behavioral guidance intervention trial; thus, the participants knew which group they were allocated to.

### 2.2. Participants

Healthy Japanese women were recruited at their 1-month postpartum medical checkup. In Japan, the medical checkups are routinely performed. Thus, almost all women visit a hospital at 1 month postpartum. Women were excluded if they were less than 20 years of age, had inadequate Japanese literacy, had diabetes, hypertension, or psychiatric diseases, followed a restricted diet due to certain diseases, or had a baby with a serious health condition.

### 2.3. Intervention

The dietary intervention was developed based on the HBM [12,13,14]. According to this model, the likelihood of adopting a healthy behavior is determined based on perceptions about the specific behavior. The main perceptions include the individual’s perceived susceptibility and severity of diseases, perceived benefits and barriers to adopting a healthy behavior. In addition, cues to action and self-efficacy have recently been added to the concepts of the model. Postpartum Japanese women do not get enough opportunities to perceive their susceptibility to diseases due to a lack of dietary assessment and feedback [2,6]. Moreover, they are less likely to receive adequate information to reduce any perceived barriers to adopting a healthier diet. Thus, we considered that the HBM framework could fit the background of postpartum Japanese women and used the model for dietary intervention. The intervention incorporated the following five steps: (1) dietary assessment; (2) individual dietary feedback; (3) dietary guidance regarding adequate intake of significant nutrients, such as protein, calcium, iron, vitamin D, and folate, as well as information on the diseases associated with the lack of these nutrients; (4) dietary guidance regarding nutrient-rich foods and easy recipes that aim to compensate for any dietary deficiencies; (5) recommendation of food groups, such as green and yellow vegetables, light-colored vegetables, lean meat, fish, eggs, and pulses. Based on the HBM [12,13,14], steps (1) and (2) in the present study were conducted for perception of susceptibility, step (3) was conducted for perception of severity of nutrition-related diseases and benefits to healthier behavior, and steps (4) and (5) were conducted to decrease perceived barriers to healthier behavior. The participants in the IG received leaflets containing information on steps (2), (3), (4), and (5) by mail, within 2 weeks of the investigations at 1 and 3 months postpartum. Dietary assessment and feedback at 3 months postpartum were conducted for external cues to action, which is a component of the HBM. Nutrients that were considered significant during the postpartum period and nutrients that postpartum Japanese women were likely to lack were the focus of the leaflet based on literature review [3]. This intervention strategy was developed with reference to previous studies of pregnant women [11,15]. The intervention was conducted by researchers who were midwives.

The CG received their individual dietary results of the three investigations (1, 3, and 6 months postpartum) and the above-mentioned leaflet by mail after the investigation at 6 months postpartum. Participants in the CG had usual care, including a home visit by public health nurses, midwives, or childcare advisors. Home visits to families with infants are required by law; thus, were conducted in both CG and IG. During home visits, mothers are often educated on dietary intake for breastfeeding and preventing anemia and constipation [16].

### 2.4. Data Collection

The participants were asked to complete a diet history questionnaire and an original questionnaire including demographic questions within 7 days of recruitment. Missing or unclear data were resolved through telephone interviews. The medical charts of the participants were reviewed to obtain information on their pregnancies and childbirths. At 3 and 6 months postpartum, participants were asked to answer another mail-in questionnaire.

### 2.5. Outcomes

Primary outcomes were nutrient intake and food group consumption at 6 months postpartum, and eventual changes in nutrient intake and food group consumption between 1 and 6 months postpartum. The secondary outcome was changes in body weight at 6 months postpartum relative to the measurement at 1 month postpartum.

### 2.6. Variables

The original questionnaire distributed to the mothers at 1 month postpartum contained questions on age, parity, education levels, household income, height, smoking habits, regular supplement usage, and infant feeding style. Maternal pre-pregnancy weight, maternal weight at 1 month postpartum, and birth weight of the infants were obtained from medical charts. Dietary intake was assessed using a brief-type self-administered diet history questionnaire (BDHQ). The BDHQ comprised a four-page fixed-portion questionnaire that assessed habitual dietary intake in the previous month and included a total of 58 food and beverage items [17,18]. Standard portion sizes for women were determined based on the National Nutrition Survey of Japan and various Japanese recipe books. Questions on consumption frequency could be answered by choosing one of seven response options ranging from “more than twice per day” to “almost never.” Dietary intakes were calculated based on the consumption frequency of selected food and beverage items, daily intake of rice and miso soup, usual cooking methods for fish and meat, and general dietary behavior. The intake of energy, protein, fat, carbohydrate, minerals (sodium, potassium, calcium, magnesium, phosphorus, iron, and zinc), and vitamins (vitamin D, α-tocopherol, vitamin B_6_, vitamin B_12_, folate, vitamin C, and β-carotene) were calculated using an ad hoc computer algorithm for the BDHQ. A validation study reported correlation coefficients of 0.26 to 0.63 between the BDHQ and semi-weighed 16-day dietary records among Japanese women for these nutrient intakes [17,18]. We calculated the energy-adjusted intake, which is the ratio of the contents of nutrients or food groups to the total energy intake, to reduce the impact of systematic misreporting on dietary intake.

Information on dietary intake, weight, and infant feeding style was collected at 3 and 6 months postpartum using questionnaires.

### 2.7. Ethical Considerations

This study was conducted according to the guidelines of the Declaration of Helsinki. The Ethics Committee of the university approved the study procedures and protocol (No. 10,536). The study was registered with the University Hospital Medical Information Network-Clinical trial registration. All of the participants provided informed written consent prior to the baseline investigation.

### 2.8. Data Analysis

The sample size was calculated with a focus on iron intake, since it is one of the most significant nutrients in the postpartum period. We estimated a total sample size of 208 women for an expected effect size of 0.40, a power of 0.80, and an alpha of 0.05, based on iron intake reported in a previous study [19]. These statistical power analyses were conducted using G-Power version 3.1. To account for potential dropouts of an estimated 30%, we aimed to recruit 296 study participants in total.

Student’s t-test, Chi-square test, or Fisher’s exact test were used to compare the baseline characteristics of the participants between the IG and CG. Mann-Whitney U tests were employed to compare the nutrient intake and food group consumption between the two groups at 1, 3, and 6 months postpartum, and to assess potential changes in these variables during the course of the study. All differences with a two-sided *p*-value < 0.05 were considered statistically significant. Statistical analyses were performed using the Statistical Package for Social Sciences version 24.0 (IBM Corp, Armonk, NY, USA).

## 3. Results

**Figure 1:**
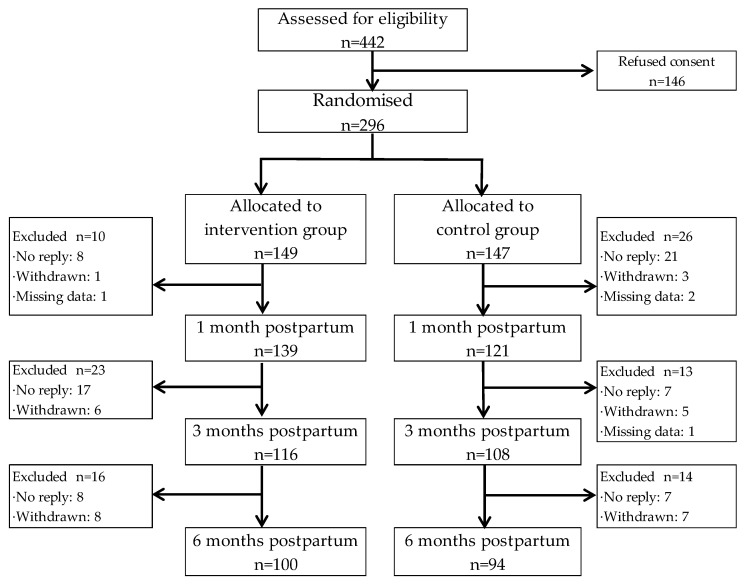
Study flow chart.

Of the 442 healthy women who were eligible at 1 month postpartum, 296 (67.0%) provided written informed consent (Figure 1). Participants were randomly allocated to either the IG (*n* = 149) or the CG (*n* = 147). Of these women, 33 did not submit questionnaires at the baseline investigation (no reply: *n* = 29, withdrawn: *n* = 4) and 3 women returned incomplete data. Thus, 260 women (139 in the IG, 121 in the CG) provided complete baseline data and were asked to answer further questionnaires. Thirty-six women were excluded at 3 months postpartum (no reply: *n* = 24, withdrawn: *n* = 11; missing data: *n* = 1), and a further 30 women were excluded at 6 months postpartum (no reply: *n* = 15, withdrawn: *n* = 15). Thus, 100 women in the IG and 94 women in the CG completed questionnaires at 6 months postpartum. No significant differences in demographic variables were found between the dropout subjects and the participants who completed all investigations (data not shown).

### 3.1. Comparison of Dietary Intake at 1 and 3 Months Postpartum between the Two Groups

Table 1 summarizes the participant characteristics at 1 month postpartum. No significant differences were observed in participant characteristics between the two groups. The levels of reported energy-adjusted nutrient intake and food group consumption at 1 month postpartum are shown in Table 2; Table 3, respectively. The energy-adjusted nutrient intakes were not significantly different between the two groups at 1 month postpartum. The median daily nutrient intake at 1 month postpartum was as follows: protein, 66.2 (interquartile range: 54.2–78.6) g/day in the IG and 69.3 (57.8–86.0) g/day in the CG; calcium, 526 (406–674) mg/day in the IG and 592 (416–762) mg/day in the CG; iron, 7.3 (5.9–8.7) mg/day in the IG and 7.9 (6.1–9.9) mg/day in the CG; vitamin D, 11.0 (7.5–15.9) μg/day in the IG and 10.5 (7.4–16.9) μg/day in the CG; and folate, 295 (234–386) μg/day in the IG and 333 (232–433) μg/day in the CG. Daily nutrient intake did not significantly differ between the IG and CG. In terms of food groups, only energy-adjusted egg consumption was significantly lower in the IG than in the CG (*p* = 0.039). No significant differences in nutrient intake and food group consumption were found between lactating and non-lactating women (data not shown).

At 3 months postpartum, only the energy-adjusted zinc intake was significantly higher in the IG than in the CG (*p* = 0.033; data not shown).

### 3.2. Comparison of Nutrient Intake at 6 Months Postpartum

Table 4 shows the differences in nutrient intake at 6 months postpartum, as well as the changes in nutrient intake from 1 to 6 months postpartum between the two groups. The energy-adjusted intakes of protein (*p* = 0.036), total dietary fiber (*p* = 0.022), potassium (*p* = 0.049), magnesium (*p* = 0.046), phosphorus (*p* = 0.024), zinc (*p* = 0.020), vitamin B_6_ (*p* = 0.022), and β-carotene (*p* = 0.047) were significantly higher in the IG than in the CG at 6 months postpartum. Changes in energy-adjusted intakes of total dietary fiber (*p* = 0.039) and iron (*p* = 0.040) from 1 to 6 months postpartum were significantly different between the two groups.

At 6 months postpartum, 88 women (88.0%) reported any types of breastfeeding, and 12 (12.0%) reported formula feeding in the IG. In the CG, 84 women (89.4%) reported any types of breastfeeding, and 10 (10.6%) reported formula feeding. No difference was found in breastfeeding rates between the two groups.

### 3.3. Comparison of Food Group Consumption at 6 Months Postpartum

Confectionery consumption was significantly lower in the IG than in the CG at 6 months postpartum (*p* = 0.002) (Table 5). In addition, changes in energy-adjusted egg consumption from 1 to 6 months postpartum were significantly different between the two groups (*p* = 0.001).

### 3.4. Comparison of Weight Changes

The mean body weight (standard deviation) was 53.4 (6.5) kg in the IG and 53.3 (7.2) kg in the CG at 3 months postpartum, and 52.7 (7.2) kg in the IG and 52.6 (7.3) kg in the CG at 6 months postpartum. There were no significant differences in the weight change from baseline to 3 or 6 months postpartum between the two groups.

## 4. Discussion

Dietary intervention based on HBM had positive effects on intake of the following nutrients in postpartum Japanese women: protein, total dietary fiber, potassium, magnesium, phosphorus, iron, zinc, vitamin B_6_, and β-carotene. Our results suggest that approaches to each perception in the HBM improved dietary behaviors during the postpartum period. In addition, second dietary feedback at 3 months postpartum might be effective as a cue to action. This was speculated from the results, showing that the intake of several nutrients at 6 months postpartum were significantly different between the two groups, despite the lack of clear differences in nutrient intake at 3 months postpartum. A dietary intervention pilot study in the United States was also conducted between 2 and 6 months postpartum and was successful in increasing intakes of dark green and deep yellow vegetables [20]. The postpartum period between 1 and 6 months postpartum could be appropriate time for dietary intervention because of increased mental allowance as time progressed after childbirth. Most women cannot afford to pay attention to good nutrition in the month following childbirth owing to preoccupation with child-rearing [21,22,23]. However, 1–2 months postpartum, women have more time to dedicate to their own health, including the preparation of well-balanced meals [23]. In addition, 50–60% of Japanese women stay with their babies at their parents’ house in the month after childbirth to receive housework support from their parents [24,25], and most return to their own houses 1–2 months postpartum and cook their meals themselves [26]. Thus, dietary intervention after 1 month postpartum could be effective in improving diet quality. On the other hand, the effects of this dietary intervention by mail were limited. Possible reasons for this might be that medical professionals could not provide direct advice to the participants and approaches to self-efficacy could not be included in our intervention. Therefore, further research examining whether adding approaches to improving self-efficacy in our intervention is more effective, is required.

The energy-adjusted iron intake at 6 months postpartum increased in more than 50% of women in the IG compared to the assessment at 1 month postpartum, while it decreased in the CG. Maternal hemoglobin recovery is often delayed, especially in lactating women with low iron intake, because the iron levels in breast milk are stable irrespective of maternal dietary intake [5]. The recommended iron intake in the lactation period is 8.5–9.0 mg/day in the absence of a menstrual cycle, and 10.5 mg/day for women whose menstruation has restarted [27]. However, more than half of our study participants did not meet these recommendations at 1 month postpartum. Our dietary intervention, including recognition of the individual iron intake and the health risks associated with iron deficiency, has the potential to achieve an increase in iron intake during the postpartum period.

Energy-adjusted intakes of protein, total dietary fiber, potassium, magnesium, phosphorus, zinc, vitamin B_6_, and β-carotene at 6 months postpartum were higher in the IG than in the CG. Although there was no significant difference in the energy intake between the two groups, significant differences in several energy-adjusted nutrient intakes would indicate an improved quality of diet. Protein was a target nutrient in this intervention because it is essential for the development of breastfed infants [28]. Proteins are present in meat, fish, and eggs, which can be taken at relative convenience without a significant adjustment to the types of foods eaten in a normal diet. Dietary feedback and the provision of a leaflet with easy recipes might be likely to motivate women in proactively consuming protein-rich foods. Magnesium, zinc, vitamin B_6_, and β-carotene are also essential for physical recovery from childbirth and maintaining an adequate supply of these nutrients in breast milk. In addition, dietary fiber is important for the prevention of constipation. Although explanations of these nutrients were not included in the intervention leaflet, the nutrients were contained in other nutrient-rich foods and recommended food groups that were introduced in the leaflet. For instance, dietary fiber is contained in a wide range of foods, such as pulses, potatoes, vegetables, fruits, and seaweeds, all of which were recommended in the leaflet. Thus, the nutrient intake might have increased as a secondary effect by consciously eating foods recommended in the leaflet. It is also possible that the leaflet generated an interest in a healthy diet in the IG, leading them to seek further information on other important nutrients and change their dietary intake. In the prenatal Japanese study [11], no nutrient intake was affected by dietary intervention based on the HBM, except for protein. However, our postpartum dietary intervention could have increased the intake of several nutrients and foods, although the effects were minor. A possible reason for the discrepancy between the results might have been the opportunity for the second dietary feedback and provision of dietary information at 3 months postpartum as a cue to action. Although further consideration would be needed, the second cue to action might have brought better outcomes.

In terms of food groups, participants in the IG consumed less confectionery at 6 months postpartum. Another randomized controlled trial for postpartum women who were overweight and obese has also shown a reduction in confectionery consumption [9]. Our intervention might have increased the health consciousness of these women, which in turn led to a lower consumption of unhealthy food items [29]. In addition, energy-adjusted egg consumption at 6 months postpartum increased in more than 50% of women in the IG compared to the assessment at 1 month postpartum, while it decreased in the CG. Egg was one of the recommended foods in our leaflets, and it is plausible that egg intake can be easily increased by intervention because eggs are inexpensive and can be easily cooked. This would lead to an increase in the intake of protein, phosphorus, and iron, which are rich in eggs.

No significant differences in changes in body weight and energy intake were found between the two groups, and the mean weights at 6 months postpartum in both groups were similar to those pre-pregnancy. Energy intake was far below the estimated energy requirement (lactating women: 2300–2350 kcal/day, non-lactating women: 1950–2000 kcal/day) in most participants, even after considering potential dietary under-reporting [27]. Japanese women tend to desire a rapid weight loss after childbirth [30], which may have also contributed to energy intake below the requirements. Nevertheless, it is essential for women to achieve sufficient energy intake for physical strength and breast milk production; thus, it is necessary to advise postpartum women to meet the recommendations for adequate energy and nutrient intakes.

The CG received only usual care in the present study. This might have made it difficult to interpret the results of the intervention, that is, to distinguish the effects of the intervention itself from the effects of the attention received by the intervention. However, we speculate that two dietary investigations at 1 and 3 months postpartum may have prompted some participants in the CG to become interested in dietary intake. In addition, all of the families with infants in Japan had home visits by public health nurses or midwives, as usual care. Home visits often include dietary guidance for mothers [16]. This opportunity might trigger an interest in their dietary intake in the CG. Thus, attention to diet may be present in both the CG and IG, and the results of the present study would reflect the effect of the intervention itself to some extent.

Our study has several limitations. Firstly, the participant characteristics may have been biased because the research was conducted at a single university hospital in an urban area. The mean age of the participants in this study was slightly higher than that of the participants in national reports [31]. Such differences in the demographic characteristics might have affected the dietary intake, although the nutritional characteristics of the participants were similar to those reported in another recent study in the postpartum period [32]. Secondly, there was higher dropout rates than expected, which may have weakened the statistical power. Thirdly, dietary intake was self-reported, and participants in the IG might unconsciously report better dietary intake at the end of the intervention because they were not blinded. In line with this, the reported dietary intake of CG might have been influenced by a newly developed interest in diet after answering the dietary questionnaires. Fourthly, the body weight at 3 and 6 months postpartum was self-reported and may differ from the actual weight. Nevertheless, we speculate that such differences would be small because a strong correlation between self-reported and measured weight has been previously reported in Japanese women [33]. Fifthly, the effects of dietary intervention on dietary intake were minor. This is partly because medical professionals could not have provided direct advice and approaches to the participants. The outcome may be even more pronounced if additional methods such as direct approaches by medical professionals were to be included in the intervention. Sixthly, we could not measure biological nutrient levels as outcomes because postpartum women did not have any routine blood tests. We did not collect blood samples for our research to prevent performing invasive procedures on the participants. Thus, we could not determine the effects of dietary interventions using objective markers. However, we had effects on self-reported dietary intake only at 6 months postpartum, although they were not observed at 3 months postpartum. In addition, we used a validated dietary questionnaire [17,18]. This may indicate that our dietary intervention had some effects on nutrient status in the postpartum period.

## 5. Conclusions

This randomized controlled trial initiated in women at 1 month postpartum indicates beneficial effects of individual dietary intervention based on HBM on the intake of protein, total dietary fiber, potassium, magnesium, phosphorus, iron, zinc, vitamin B_6_, and β-carotene at 6 months postpartum. Such dietary intervention would help to improve dietary intake of postpartum women in clinical settings, although the observed effects were comparatively limited. Further intervention research, which adds other components, such as increasing self-efficacy in changing dietary habits, is required to enhance the effects.

## Figures and Tables

**Table 1:** Participant characteristics at 1 month postpartum.

		Intervention Group (n = 139)		Control Group (n = 121)		*p*
		Mean ± SD or n (%)		Mean ± SD or n (%)		
Age [year]		35.4	±4.7	35.2	±4.5	0.694 ^†^
Parity: Primiparae [n (%)]		83	(59.7)	76	(62.7)	0.609
Education level [n (%)]						
	High school	9	(6.5)	9	(7.4)	0.680
	Junior or technical college	36	(25.9)	34	(28.1)	
	University or above	94	(67.6)	78	(64.4)	
Annual household income [n (%)]						
	< 5 million Japanese yen	19	(13.6)	11	(9.1)	0.475
	5–9 million Japanese yen	52	(37.4)	54	(44.6)	
	> 9 million Japanese yen	68	(48.9)	56	(46.3)	
Height [cm]		158.7	±5.5	158.7	±5.5	0.750 ^†^
Pre-pregnancy weight [kg]		52.2	±7.6	52.1	±7.6	0.966 ^†^
Gestational weight gain [kg]		9.6	±3.3	9.6	±3.2	0.974 ^†^
Weight at 1 month postpartum [kg]		54.6	±7.3	54.1	±7.2	0.642 ^†^
BMI at 1 month postpartum [kg/m^2^]		21.6	±2.5	21.4	±2.5	0.485 ^†^
	Underweight (BMI < 18.5) [n (%)]	6	(4.3)	8	(6.6)	0.677
	Normal (18.5 ≤ BMI < 25.0) [n (%)]	121	(87.7)	105	(86.8)	
	Overweight / Obesity (BMI > 25.0) [n (%)]	11	(8.0)	8	(6.6)	
Regular supplement usage [n (%)]		54	(38.8)	50	(41.3)	0.685
	Folic acid supplements [n (%)]	30	(21.6)	22	(18.2)	0.542
	Multivitamins [n (%)]	10	(7.2)	11	(9.1)	0.697
	Vitamin C supplements [n (%)]	10	(7.2)	4	(3.3)	0.673
	Iron supplements [n (%)]	28	(20.1)	27	(22.3)	0.987
Breastfeeding [n (%)]		132	(95.0)	118	(97.5)	0.346 ^‡^
Birth weight (infant) [g]		2948	±373	3034	±373	0.064 ^†^

BMI, body mass index; SD, standard deviation. Chi-square test. ^†^ Student’s *t*-test. ^‡^ Fisher’s exact test. Intervention group: Individual dietary intervention based on the health belief model at 1 and 3 months postpartum (dietary assessment, individual feedback, and dietary guidance). Control group: Usual care (including home visits by public health nurses or midwives, etc.

**Table 2:** Nutrient intake at 1 month postpartum.

		Intervention Group (n = 139)		Control Group (n = 121)		*p*
		Median (Interquartile Range)		Median (Interquartile Range)		
Energy [kcal/day]		1703	(1443–1995)	1868	(1558–2109)	0.090
Protein [% energy]		15	(14–17)	15	(14–17)	0.845
Fat [% energy]		29	(25–33)	29	(26–31)	0.709
	Omega-3 PUFA [% energy]	1.3	(1.1–1.5)	1.2	(1.0–1.5)	0.297
	EPA [% energy]	0.13	(0.09–0.18)	0.12	(0.08–0.18)	0.425
	DHA [% energy]	0.22	(0.16–0.30)	0.21	(0.16–0.30)	0.607
	Omega-6 PUFA [% energy]	5.2	(4.6–6.1)	5.3	(4.5–5.9)	0.935
Hydrocarbonate [% energy]		54	(50–59)	55	(51–59)	0.445
Total dietary fiber [g/1000 kcal]		6.8	(5.9–8.1)	6.8	(5.7–8.2)	0.968
Sodium [mg/1000 kcal]		2125	(1875–2483)	2115	(1816–2435)	0.740
Potassium [mg/1000 kcal]		1393	(1151–1644)	1389	(1185–1619)	0.827
Calcium [mg/1000 kcal]		307	(247–380)	328	(263–396)	0.328
Magnesium [mg/1000 kcal]		132	(115–155)	133	(116–154)	0.760
Phosphorus [mg/1000 kcal]		572	(507–666)	583	(526–655)	0.692
Iron [mg/1000 kcal]		4.3	(3.6–4.9)	4.2	(3.7–5.0)	0.861
Zinc [mg/1000 kcal]		4.6	(4.2–5.0)	4.6	(4.2–5.0)	0.743
Vitamin D [μg/1000 kcal]		6.2	(4.7–9.2)	6.0	(4.3–8.5)	0.359
α-tocopherol [mg/1000 kcal]		4.3	(3.8–4.9)	4.2	(3.8–4.9)	0.878
Vitamin B_6_ [mg/1000 kcal]		0.7	(0.6–0.8)	0.7	(0.6–0.8)	0.464
Vitamin B_12_ [μg/1000 kcal]		4.3	(3.2–6.0)	4.2	(3.3–5.8)	0.956
Folate [μg/1000 kcal]		177	(139–216)	172	(141–223)	0.821
Vitamin C [mg/1000 kcal]		60	(45–75)	61	(47–78)	0.494
β-carotene [μg/1000 kcal]		2069	(1447–2746)	1982	(1225–2692)	0.529

DHA, docosahexaenoic acids; EPA, eicosapentaenoic acids; PUFA, polyunsaturated fatty acids. Mann-Whitney *U* test. Intervention group: Individual dietary intervention based on the health belief model at 1 and 3 months postpartum (dietary assessment, individual feedback, and dietary guidance). Control group: Usual care (including home visits by public health nurses or midwives, etc.

**Table 3:** Food consumption at 1 month postpartum.

	Intervention Group (n = 139)		Control Group (n = 121)		*p*
	Median (Interquartile range)		Median (Interquartile range)		
Cereals [g/1000kcal]	215	(170–266)	209	(170–247)	0.306
Pulses [g/1000kcal]	33	(20–54)	39	(23–53)	0.348
Potatoes [g/1000kcal]	28	(14–38)	27	(14–39)	0.753
Sugar [g/1000kcal]	2.3	(1.3–3.2)	2.1	(1.3–3.1)	0.831
Confectioneries [g/1000kcal]	25	(15–38)	29	(17–44)	0.068
Oil [g/1000kcal]	5.5	(4.0–7.3)	5.5	(3.9–6.9)	0.671
Fruits [g/1000kcal]	59	(32–86)	65	(33–96)	0.357
Green and yellow vegetables [g/1000kcal]	70	(50–94)	68	(50–95)	0.982
Other vegetables [g/1000kcal]	82	(64–111)	83	(62–106)	0.798
Fish and shellfish [g/1000kcal]	36	(25–46)	34	(22–48)	0.433
Meat [g/1000kcal]	41	(31–51)	38	(32–46)	0.117
Eggs [g/1000kcal]	16	(11–28)	22	(13–30)	0.039
Dairy products [g/1000kcal]	87	(43–129)	94	(43–138)	0.582

Mann-Whitney *U* test. Intervention group: Individual dietary intervention based on the health belief model at 1 and 3 months postpartum (dietary assessment, individual feedback, and dietary guidance). Control group: Usual care (including home visits by public health nurses or midwives, etc.).

**Table 4:** Changes in nutrient intake from 1 month to 6 months postpartum.

		Nutrient Intake at 6 Months Postpartum					Changes in Nutrient Intake from 1 Month to 6 Months Postpartum				
		Intervention Group (n = 100)		Control Group (n = 94)		*p*	Intervention Group (n = 100)		Control Group (n = 94)		*p*
		Median (Interquartile Range)		Median (Interquartile Range)			Median (Interquartile Range)		Median (Interquartile Range)		
Energy [kcal/day]		1678	(1357–2007)	1679	(1441–1924)	0.894	−77	(−366–189)	−142	(−397–105)	0.417
Protein [% energy]		16	(14–18)	15	(14–16)	0.036	0.6	(−0.9–2.5)	0.1	(−1.4–1.6)	0.160
Fat [% energy]		28	(25–32)	29	(25–32)	0.943	0.2	(−4.1–3.2)	0.5	(−3.9–3.8)	0.848
	Omega-3 PUFA [% energy]	1.3	(1.0–1.5)	1.2	(1.0–1.4)	0.177	0.1	(−0.2–0.3)	0.0	(−0.2–0.3)	0.452
	EPA [% energy]	0.13	(0.09–0.20)	0.12	(0.09–0.15)	0.169	0.00	(−0.04–0.06)	0.01	(−0.04–0.04)	0.611
	DHA [% energy]	0.22	(0.16–0.33)	0.21	(0.16–0.26)	0.145	0.00	(−0.08–0.11)	0.00	(−0.07–0.06)	0.450
	Omega-6 PUFA [% energy]	5.2	(4.4–6.1)	5.5	(4.8–6.0)	0.444	0.1	(-0.7–0.9)	0.1	(−0.7–1.0)	0.963
Hydrocarbonate [% energy]		54	(49–58)	56	(51–60)	0.289	−1	(−5–4)	−1	(−5–4)	0.784
Total dietary fiber [g/1000 kcal]		7.3	(6.1–8.4)	6.6	(5.8–7.9)	0.022	0.0	(−0.8–0.8)	0.0	(−1.2–1.0)	0.039
Sodium [mg/1000 kcal]		2149	(1822–2452)	2127	(1744–2457)	0.718	−3	(−315–321)	18	(−336–285)	0.529
Potassium [mg/1000 kcal]		1456	(1191–1703)	1329	(1149–1559)	0.049	57	(−177–268)	9	(−247–218)	0.098
Calcium [mg/1000 kcal]		326	(258–402)	297	(247–364)	0.074	6	(−52–73)	−8	(−70–43)	0.088
Magnesium [mg/1000 kcal]		141	(121–160)	130	(116–150)	0.046	9	(−8–24)	0	(−16–17)	0.052
Phosphorus [mg/1000 kcal]		596	(539–669)	566	(510–630)	0.024	13	(−40–96)	−10	(−80–60)	0.056
Iron [mg/1000 kcal]		4.4	(3.7–5.3)	4.1	(3.7–4.8)	0.072	0.2	(−0.5–0.8)	−0.1	(−0.9–0.5)	0.040
Zinc [mg/1000 kcal]		4.7	(4.4–5.2)	4.5	(4.2–4.8)	0.020	0.1	(−0.3–0.5)	−0.1	(−0.4–0.3)	0.053
Vitamin D [μg/1000 kcal]		6.1	(4.4–10.4)	5.7	(4.2–8.4)	0.171	0.0	(−1.8–2.0)	0.0	(−1.7–2.0)	0.650
α-tocopherol [mg/1000 kcal]		4.2	(3.6–4.9)	4.2	(3.8–4.7)	0.806	0.1	(−0.6–0.7)	0.1	(−0.7–0.7)	0.632
Vitamin B_6_ [mg/1000 kcal]		0.7	(0.6–0.8)	0.7	(0.6 - 0.8)	0.022	0.0	(−0.1–0.1)	0.0	(−0.1–0.1)	0.293
Vitamin B_12_ [μg/1000 kcal]		4.4	(3.2–6.1)	4.1	(3.3–5.4)	0.321	0.1	(−1.4–1.2)	−0.4	(−1.4–1.2)	0.553
Folate [μg/1000 kcal]		185	(141–228)	172	(148–206)	0.258	11	(−33–50)	2	(−48–33)	0.172
Vitamin C [mg/1000 kcal]		59	(45–79)	58	(46–67)	0.279	1	(−10–19)	1	(−18–11)	0.201
β-carotene [μg/1000 kcal]		2037	(1561–3016)	1805	(1330–2530)	0.047	217	(−584–882)	28	(−682–651)	0.292

DHA, docosahexaenoic acids; EPA, eicosapentaenoic acids; PUFA, polyunsaturated fatty acids. Mann-Whitney *U* test. Intervention group: Individual dietary intervention based on the health belief model at 1 and 3 months postpartum (dietary assessment, individual feedback, and dietary guidance). Control group: Usual care (including home visits by public health nurses or midwives, etc.).

**Table 5:** Changes in food consumption from 1 month to 6 months postpartum.

	Food Consumption at 6 Months Postpartum					Changes in Food Consumption from 1 Month to 6 Months Postpartum				
	Intervention Group (n = 100)		Control Group (n = 94)		*p*	Intervention Group (n = 100)		Control Group (n = 94)		*p*
	Median (Interquartile Range)		Median (Interquartile Range)			Median (Interquartile Range)		Median (Interquartile Range)		
Cereals [g/1000kcal]	215	(174–253)	206	(168–254)	0.596	−1	(−45–34)	3	(−48–48)	0.692
Pulses [g/1000kcal]	37	(24–55)	39	(21–56)	0.820	5	(−7–17)	1	(−11–12)	0.182
Potatoes [g/1000kcal]	30	(15–42)	27	(13–38)	0.460	1	(−12–13)	1	(−14–16)	0.914
Sugar [g/1000kcal]	1.8	(1.1–2.6)	1.9	(1.1–2.9)	0.784	−0.2	(−1.4–0.8)	−1.1	(−0.1–0.8)	0.978
Confectioneries [g/1000kcal]	24	(14–36)	33	(23–45)	0.002	1	(−10–13)	2	(−10–14)	0.732
Oil [g/1000kcal]	5.6	(4.4–7.4)	6.1	(4.7–7.3)	0.457	0.2	(−1.6–1.8)	0.5	(−1.8–2.1)	0.541
Fruits [g/1000kcal]	55	(31–88)	49	(29–83)	0.551	−6	(−28 –16)	−12	(−41–16)	0.258
Green and yellow vegetables [g/1000kcal]	64	(44–92)	59	(41–85)	0.143	−2	(−23–21)	−5	(−33–17)	0.249
Other vegetables [g/1000kcal]	86	(66–119)	80	(55–108)	0.156	6	(−15–30)	0	(−29–26)	0.278
Fish and shellfish [g/1000kcal]	35	(24–59)	33	(24–44)	0.257	1	(−13–15)	0	(−12–12)	0.677
Meat [g/1000kcal]	40	(32–51)	41	(30–53)	0.850	1	(−13–8)	1	(−11–16)	0.217
Eggs [g/1000kcal]	19	(12–28)	15	(7–28)	0.094	1	(−5–9)	−2	(−12–3)	0.001
Dairy products [g/1000kcal]	87	(47–137)	87	(45–118)	0.455	3	(−34–37)	0	(−49–28)	0.526

Mann-Whitney *U* test. Intervention group: Individual dietary intervention based on the health belief model at 1 and 3 months postpartum (dietary assessment, individual feedback, and dietary guidance). Control group: Usual care (including home visits by public health nurses or midwives, etc.).

## Data Availability

Not applicable.
